# Comparing COVID-19 risk factors in Brazil using machine learning: the importance of socioeconomic, demographic and structural factors

**DOI:** 10.1038/s41598-021-95004-8

**Published:** 2021-08-02

**Authors:** Pedro Baqui, Valerio Marra, Ahmed M. Alaa, Ioana Bica, Ari Ercole, Mihaela van der Schaar

**Affiliations:** 1 Núcleo de Astrofísica e Cosmologia, Universidade Federal do Espírito Santo, Vitória, ES Brazil; 2 Departamento de Física, Universidade Federal do Espírito Santo, Vitória, ES Brazil; 3 Department of Electrical and Computer Engineering, University of California Los Angeles, Los Angeles, CA USA; 4 Department of Engineering Science, University of Oxford, Oxford, UK; 5 The Alan Turing Institute, London, UK; 6 Department of Medicine, University of Cambridge, Cambridge, UK; 7 Cambridge Centre for Artificial Intelligence in Medicine, Cambridge, UK; 8 Department of Applied Mathematics and Theoretical Physics and Department of Population Health, University of Cambridge, Cambridge, UK

**Keywords:** Health policy, Viral infection, Epidemiology

## Abstract

The COVID-19 pandemic continues to have a devastating impact on Brazil. Brazil’s social, health and economic crises are aggravated by strong societal inequities and persisting political disarray. This complex scenario motivates careful study of the clinical, socioeconomic, demographic and structural factors contributing to increased risk of mortality from SARS-CoV-2 in Brazil specifically. We consider the Brazilian SIVEP-Gripe catalog, a very rich respiratory infection dataset which allows us to estimate the importance of several non-laboratorial and socio-geographic factors on COVID-19 mortality. We analyze the catalog using machine learning algorithms to account for likely complex interdependence between metrics. The XGBoost algorithm achieved excellent performance, producing an AUC-ROC of 0.813 (95% CI 0.810–0.817), and outperforming logistic regression. Using our model we found that, in Brazil, socioeconomic, geographical and structural factors are more important than individual comorbidities. Particularly important factors were: The state of residence and its development index; the distance to the hospital (especially for rural and less developed areas); the level of education; hospital funding model and strain. Ethnicity is also confirmed to be more important than comorbidities but less than the aforementioned factors. In conclusion, socioeconomic and structural factors are as important as biological factors in determining the outcome of COVID-19. This has important consequences for policy making, especially on vaccination/non-pharmacological preventative measures, hospital management and healthcare network organization.

## Introduction

The COVID-19 pandemic is having a particularly devastating impact on Brazil with, at the time of writing, half a million registered cumulative deaths, second only to the USA^1^. Brazil’s social, health and economic crises are aggravated by strong societal inequities^2^ and political disarray^3^. COVID-19 outcomes are likely to be the result of the interplay between patient and environmental factors. Age is now well established as the dominant determinant of mortality^4–7^. We have previously demonstrated the important effect of ethnicity and socioeconomic status in determining outcome in Brazil^2^. A number of institutional and organizational effects may also be important. It has been shown that treatment site seems to have a substantial association with mortality, comparable to the effect of comorbidity, at least for intensive care outcomes^8^. This suggests that institutional and organizational factors may be important. This is reasonable as it is likely that different hospitals may vary in their ability to respond to a surge in cases either because they are locally overwhelmed, experience an early influx of patients before surge capacity can be put into place or because they are inherently less able to expand capacity. A limited number of studies have attempted to look at this. A recent study in the United States did find evidence to support an association between hospital strain and increased mortality^9^ for critical care patients, but not ward patients, and that this relationship changed over time. A similar negative impact of intensive care capacity was seen in Belgium^10^. A full understanding of the interplay between patient and healthcare system factors is crucial for rational, dynamic allocation of hospital resources as well as the targeting of both pharmacological and non-pharmacological interventions. Healthcare systems vary substantially around the world, making local evaluation important. To our knowledge, this has not previously been undertaken in Brazil. Healthcare organizational factors are likely to be, to some extent, co-linear with other socioeconomic predictors and their effects may be non-linear: The extent to which organizational effects are real or the result of a failure to completely adjust for other factors in a linear model is not known. This observation motivates the use of explainable machine learning models able to deal with complex interactions and non-linear relationships.

**Figure 1:**
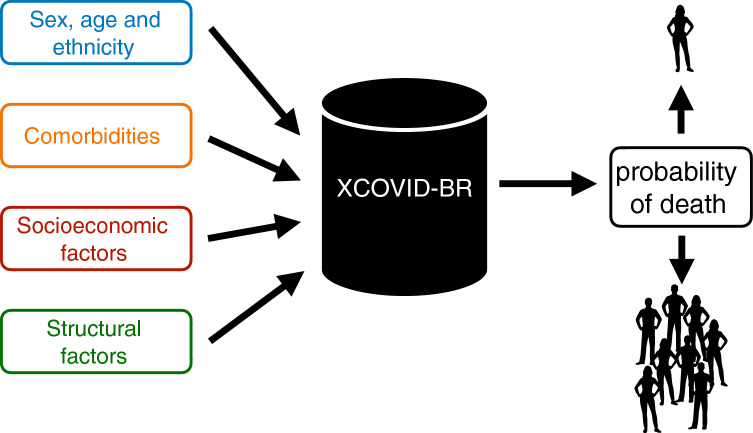
The XCOVID-BR machine learning model. XCOVID-BR takes as input a range of medical, socioeconomic and structural factors and returns as output the probability of death by COVID-19. XCOVID-BR can be applied to individuals, groups or whole sections of the Brazilian population.

In this study, we use the Brazilian SIVEP-Gripe respiratory infection surveillance dataset^11^ to study demographic, patient, socioeconomic and organizational structure influences on COVID-19 outcome. As depicted in Fig. 1, we model the linear and nonlinear correlations among the covariates using the successful XGBoost machine learning technique. We name ‘XCOVID-BR’ the XGBoost model that achieves the highest performance. The goal of this work is to provide the scientific community and, in particular, the Brazilian authorities with a ranking of the most important social, health and economic risk factors.

## Materials and methods

**Figure 2:**
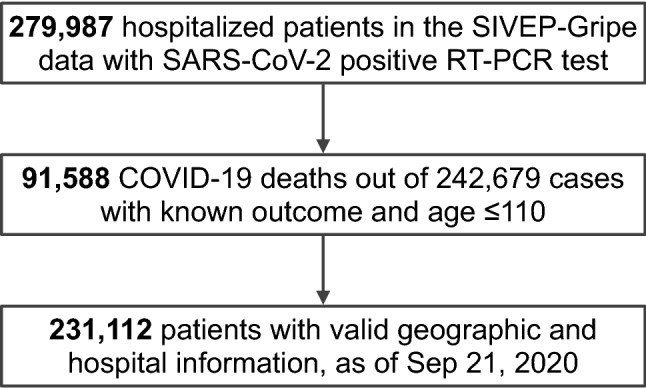
Flowchart of SIVEP-Gripe data used in this study. SARS-CoV-2 stands for severe acute respiratory syndrome coronavirus 2. SIVEP-Gripe stands for *Sistema de Informação de Vigilância Epidemiológica da Gripe*.

We analyze COVID-19 hospital mortality using the public SIVEP-Gripe dataset (Sistema de Informação de Vigilância Epidemiológica da Gripe), a prospectively collected respiratory infection registry which is maintained by the Brazilian Ministry of Health for the purposes of recording cases of severe acute respiratory syndrome (SARS) across both public and private hospitals^11^. We analyze data collected from February 25 to September 21, 2020. Out of the 279,987 hospitalized patients that had a positive RT-PCR test for SARS-CoV-2, 242,679 cases have known outcome and age $\le$110. We consider only patients who were admitted to hospital in order to be less sensitive to the regional variability of testing. Finally, as we are interested also in socioeconomic factors, we restrict our analysis to the 231,112 patients whose files contain geographic information and type of healthcare (public or private). See Fig. 2.

We initially consider 30 patient features including clinical (age, sex, ethnicity, comorbidities and symptoms), socio-geographic (education, state, municipal human development index MHDI, city type) and structural hospital-level (distance from patient to hospital, time-dependent strain and funding) factors. In order to capture the time-varying pressure on individual hospitals, we defined ‘hospital strain’ as the number of hospitalized patients during the admission week divided by a metric of hospital capacity. As capacity numbers data were not available for all the hospitals considered, we used as a proxy the total number of hospitalizations according to the 2019 SIVEP-Gripe dataset. The 231,112 patients that we consider come from 1,801 different cities and from 3,991 different hospitals. This richness allows us to disentangle the importance of a factor from the one of its covariates, fully considering all the correlations.

The prediction task was formulated as a binary classification problem for hospital mortality, with 0 representing death and 1 representing recovery. The analysis was performed using XGB but Logistic Regression, K-Nearest Neighbors, Neural Network, Random Forest and Support Vector Machine algorithms were also evaluated and are included in the Supplementary Materials for completeness (Section S3D). Our models are implemented in Python through the scikit-learn and XGB packages^12,13^.

For the training and test sets, we used 80% (184,889 patients) and 20% (46,223 patients) random split with $k=10$-fold cross validation. As metrics we consider the area under the receiver-operating characteristic curve (AUC-ROC) and the average precision (AP), which is the area under the precision-recall curve relative to a given classification (recovery or death). Feature importance is analyzed using the permutation method: the relationship between feature and target is broken via a random shuffle and feature importance is defined as the corresponding decrease in the AUC-ROC metric (see the Supplementary Materials, Section S3E, for more details and robustness tests).

The SIVEP-Gripe catalog has missing values. In the case of comorbidities or symptoms we imputed missing values as the clinical feature being absent for the individual^2^. For the remaining variables we did not perform pre-processing for the XGB algorithm as the latter already imputes missing data. A table with the percentages of patients with missing values is available in the Supplementary Materials (Section S3C).

The study was conducted and reported in line with the Transparent Reporting of a multivariable prediction model for Individual Prognosis Or Diagnosis (TRIPOD)^14^.

## Results

**Figure 3:**
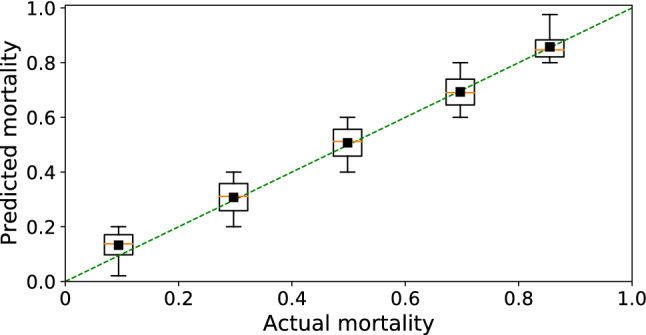
Model calibration. The calibration analysis shows that mortality predicted by the XCOVID-BR model performs uniformly across bins of mortality.

Our ‘XCOVID-BR’ XGBoost model achieved the highest performance of the models tested and is considered in the subsequent analysis. The model is publicly available at https://github.com/PedroBaqui/XCOVID-BR. We found that the XGBoost algorithm achieves excellent performance of AUC-ROC=0.813 (95% CI 0.810–0.817) compared to the logistic regression’s AUC-ROC=0.766 (95% CI 0.761–0.770, full comparison table in the Supplementary Materials, Section S3D). Model calibration is shown in Fig. 3. While the difference in AUC-ROC between XGBoost and logistic regression may seem not large, it is in fact significant. For instance, holding specificity at 80.0%, XGBoost correctly predicted death by COVID-19 (positive condition) for 1,466 more patients (8.4% of the 17,357 patients) and, holding sensitivity at 80%, XGBoost correctly predicted survival for 1,971 more patients (6.8% of the 28,866 patients), in comparison with logistic regression. Equivalently, at 80% of specificity, XGBoost and logistic regression featured a sensitivity of 64.7% and 56.2%, respectively, while at 80% of sensitivity, they featured a specificity of 66.5% and 59.7%, respectively.

**Figure 4:**
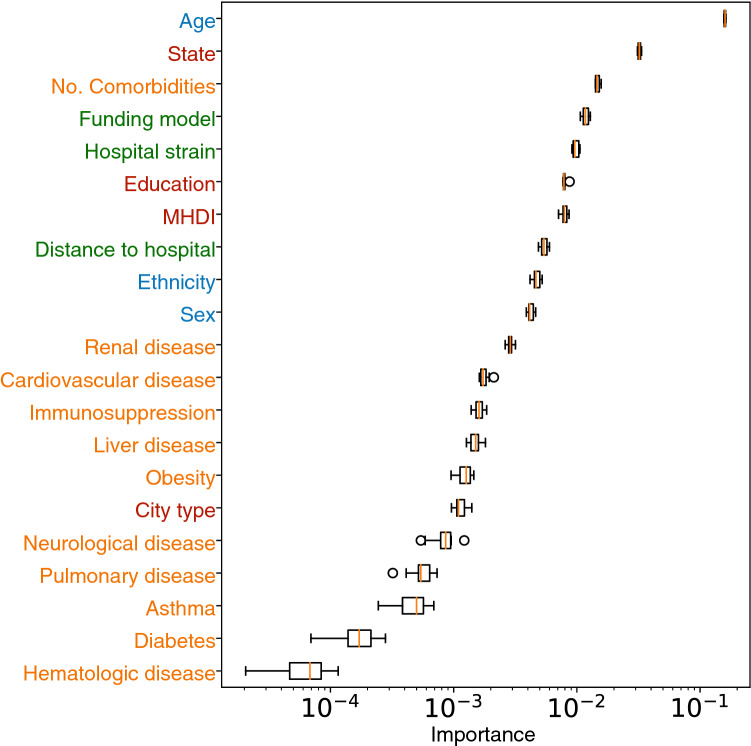
Relative feature importance (median and IQR) for mortality risk to COVID-19. The coloring marks the categories listed in Fig. 1. Feature importance is estimated via the permutation method and a logarithmic scale is employed for clarity.

**Figure 5:**
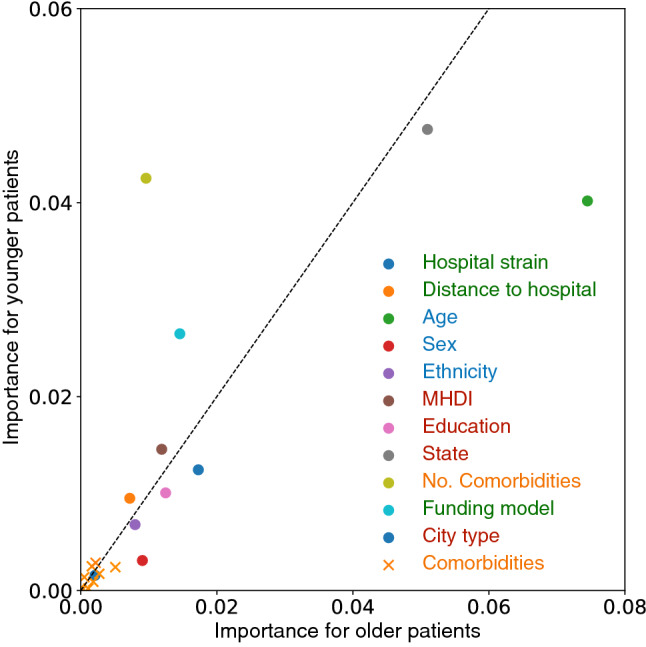
Feature importance for older and younger patients. Each point represents a feature in the SIVEP-Gripe dataset, and the axes show the relative importance for COVID-19 mortality prediction for older ($\ge$60 years, *x* axis) and younger ($<60$ years, *y* axis) hospitalized patients. Variables deviating from the dotted identity line suggests a different relative importance for the groups. The coloring marks the categories listed in Fig. 1.

Figure 4 shows the importance of the features considered in this analysis, excluding symptoms as they are not related to the patients’ pre-infection conditions. The XCOVID-BR model shows that, in Brazil, comorbidities showed less association with outcome than socioeconomic, structural and ethnic factors, and confirms the well-known importance of age.

In Fig. 5 we again use the permutation method, but we split the test set between younger ($<60$ years, AUC-ROC=0.770, 95% CI 0.763–0.777) and older ($\ge$60 years, AUC-ROC=0.717, 95% CI 0.711–0.725) patients, 24,277 and 21,946 patients respectively (in Brazil patients over 60 are considered elderly^2^). We find that for younger patients the state and the number of comorbidities play a more important role than their own age (Fig. 5). On the other hand, for elderly patients state has a greater importance and variations in age within this group are disproportionately important.

**Figure 6:**
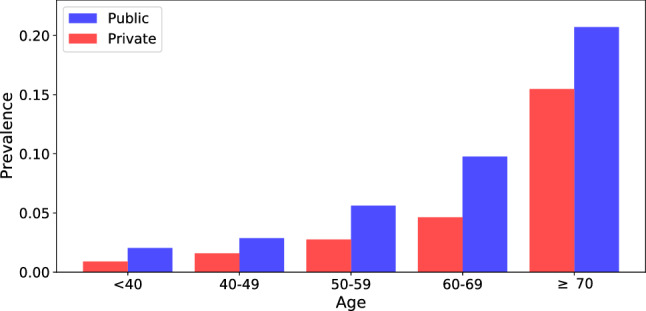
Mortality rate for public and privately funded hospitals, stratified according to age. The bars are normalized by dividing the fatalities by the total number of cases for each type of hospital funding model.

As seen from the analysis of Fig. 4, hospital funding model (private or public) is an important feature. Figure 6 shows the mortality rate for patients admitted to public and private hospitals, stratified according to age (the dominant factor associated with outcome). For all the age bins, mortality was consistently higher in public hospitals.

**Table 1:** Demographic and socio-geographic characteristics and coexisting conditions among survivors and non-survivors of COVID-19. Data are *n* (%) or mean (SD). The AUC-ROC value is relative to the XGB algorithm for patients belonging to each category. We also show the odds ratios and statistical significance for mortality (univariate comparisons).

	Survivors	Non-survivors	AUC (95% CI)	OR (95% CI)	p value
Brazil	143889(62.3%)	87223(37.7%)	0.813[0.810, 0.817]	–	–
Age (years)	54.3 ± 17.7	68.6± 15.5	–	–	–
**Sex**					
Women	63919(63.5%)	36700(36.5%)	0.811 [0.805, 0.816]	–	–
Men	79941(61.3%)	50511(38.7%)	0.815 [0.810, 0.820]	1.100 [1.082, 1.119]	<0.001
**Funding model**					
Private	35830(74.7%)	12148(25.3%)	0.843[0.832, 0.851]	–	–
Public	108059 (59.0%)	75075(41.0%)	0.799 [0.795, 0.802]	2.049 [2.003, 2.096]	<0.001
**MHDI**					
Very High	63888(70.0%)	27430(30.0%)	0.816[0.810, 0.823]	0.708 [0.697, 0.720]	<0.001
High	72657(58.5%)	51552(41.5%)	0.802[0.797, 0.808]	1.170 [1.154, 1.187]	<0.001
Medium-Low	7344 (47.1%)	8241(52.9%)	0.791[0.776, 0.809]	1.851 [1.792, 1.912]	<0.001
**Ethnic group**					
White	55040 (64.1%)	30874(35.9%)	0.813 [0.806, 0.821]	0.866 [0.851, 0.881]	<0.001
Black	6585(57.5%)	4871(42.5%)	0.803 [0.780, 0.816]	1.142 [1.099, 1.186]	<0.001
East Asian	1521(60.2%)	1005(39.8%)	0.789 [0.742, 0.825]	1.020 [0.941, 1.105]	0.632
Brown	38700 (57.0%)	29205(43.0%)	0.804 [0.797, 0.812]	1.165 [1.144, 1.186]	<0.001
Indigenous	280 (56.6%)	215(43.4%)	0.891[0.821, 0.948]	1.185 [0.992, 1.416]	0.061
**Macro-regions**					
North	5974(49.1%)	6192 (50.9%)	0.781[0.765, 0.796]	1.710 [1.649, 1.773]	<0.001
Northeast	21830 (51.7%)	20421(48.3%)	0.800[0.791, 0.807]	1.543 [1.511, 1.576]	<0.001
Central-West	11877 (63.9%)	6724 (36.4%)	0.810[0.796, 0.824]	0.934 [0.905, 0.963]	<0.001
Southeast	84551 (65.4%)	44796(34.6%)	0.811[0.805, 0.816]	0.874 [0.862, 0.887]	<0.001
South	19657 (68.4%)	9090 (31.6%)	0.809[0.800, 0.822]	0.763 [0.743, 0.783]	<0.001
**City type**					
Urban	124635 (62.6%)	74523 (37.4%)	0.813 [0.808, 0.818]	0.988 [0.976, 1.001]	0.067
Peri-Urban	382 (54.7%)	316 (45.3%)	0.795 [0.711, 0.859]	1.367 [1.178, 1.587]	<0.001
Rural	3288 (54.0%)	2797(46.0%)	0.798 [0.769, 0.823]	1.406 [1.336, 1.480]	<0.001
**Education level**					
Illiterate	2048(39.3%)	3169(60.7%)	0.762[0.733, 0.790]	2.558 [2.415, 2.709]	<0.001
Elem. school I	10912 (51.0%)	10499(49.0%)	0.773[0.759, 0.785]	1.590 [1.543, 1.639]	<0.001
Elem. school II	9026(59.1%)	6242(40.9%)	0.808[0.793, 0.824]	1.143 [1.104, 1.184]	<0.001
High school	18244 (71.0%)	7459(29.0%)	0.828[0.817, 0.840]	0.676 [0.656, 0.697]	<0.001
Higher education	9603(77.6%)	2778(22.4%)	0.874[0.860, 0.889]	0.478 [0.457, 0.500]	<0.001
**Comorbidities**					
Cardiovascular d.	43068 (53.1%)	37983 (46.9%)	0.767[0.760, 0.774]	1.821 [1.789, 1.853]	<0.001
Asthma	4453 (68.8%)	2021 (31.2%)	0.810[0.781, 0.837]	0.749 [0.710, 0.790]	<0.001
Diabetes	31719 (52.2%)	29046 (47.8%)	0.758[0.750, 0.766]	1.779 [1.745, 1.812]	<0.001
Pulmonary dis.	4007 (42.1%)	5500 (57.9%)	0.746[0.723, 0.772]	2.369 [2.272, 2.469]	<0.001
Obesity	7161 (59.0%)	4970 (41.0%)	0.771[0.754, 0.793]	1.167 [1.125, 1.212]	<0.001
Immunosuppr.	3407 (47.3%)	3795 (52.7%)	0.741[0.715, 0.766]	1.891 [1.804, 1.982]	<0.001
Renal dis.	4112 (38.3%)	6632 (61.7%)	0.719[0.699, 0.738]	2.818 [2.708, 2.933]	<0.001
Liver dis.	925 (40.2%)	1377 (59.8%)	0.731[0.695, 0.773]	2.499 [2.298, 2.717]	<0.001
Neurological dis.	3905 (39.2%)	6068(60.8%)	0.727[0.701, 0.753]	2.701 [2.592, 2.815]	<0.001
Hematologic dis.	1054 (50.5%)	1035(49.5%)	0.804[0.767, 0.851]	1.640 [1.505, 1.788]	<0.001
**Symptoms**					
Fever	99656(65.1%)	53497(34.9%)	0.813[0.808, 0.818]	0.719 [0.706, 0.732]	<0.001
Vomiting	12269(67.0%)	6049(33.0%)	0.810[0.793, 0.826]	0.812 [0.786, 0.838]	<0.001
Cough	110183 (65.0%)	59246(35.0%)	0.808[0.804, 0.813]	0.665 [0.653, 0.678]	<0.001
Sore throat	28238(68.5%)	12979(31.5%)	0.821[0.812, 0.831]	0.729 [0.712, 0.745]	<0.001
Respiratory disc.	74879(56.8%)	56912(43.2%)	0.803[0.798, 0.808]	1.767 [1.737, 1.799]	<0.001
Shortness breath	96954(58.8%)	68033(41.2%)	0.802[0.797, 0.806]	1.771 [1.736, 1.807]	<0.001
Diarrhea	21837(69.1%)	9746(30.9%)	0.811[0.799, 0.820]	0.714 [0.696, 0.733]	<0.001
SpO2< 95%	75083(55.2%)	61021(44.8%)	0.787[0.780, 0.795]	2.209 [2.169, 2.249]	<0.001

Table 1 shows the demographic and socio-geographic characteristics and coexisting conditions among survivors and non-survivors. We also show the AUC-ROC relative to the XGB algorithm for patients belonging to each category, the odds ratio (OR) and the corresponding *p*-values. Within categories, we find qualitative agreement between the OR values and the relative feature importance shown in Fig. 4. However, OR and feature importance weigh differently the various categories. For example, the OR analysis gives more significance to comorbidities. This highlights the beneficial use of the XGB model in coping with correlations between the covariates.

**Figure 7:**
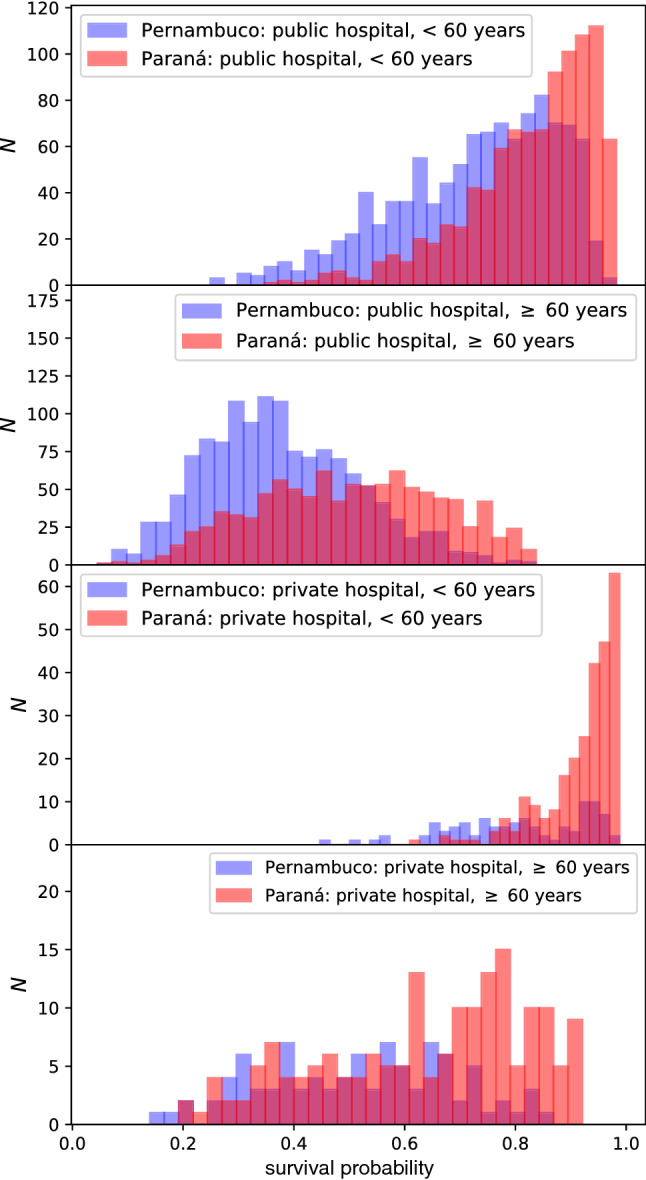
Distribution of survival probability—ranging from 0 (death) to 1 (recovery)—as estimated by the XCOVID-BR model. We contrast typical publicly and privately funded hospitals from Pernambuco, an example of a region in the more socioeconomically challenged Northeast, with examples from the richer Paraná region in the South. Stratifying by age, the dominant *clinical* predictor of mortality, it is apparent that the probability distribution is skewed with lower mortality in the wealthier (Paraná) region and this is particularly apparent in younger patients and in privately-funded hospitals.

Finally, we adopt the XCOVID-BR model in order to estimate the mortality risk of specific sections of the Brazilian population. Given a patient’s non-laboratorial data, the XCOVID-BR model returns a probability ranging from 0 (death) to 1 (recovery). One can then estimate the overall risk of a group by studying the distribution of the XCOVID-BR outcomes. Figure 7 shows the XCOVID-BR model applied to age and hospital subgroups taken from the states of Pernambuco (Northeast) and Paraná (South).

## Discussion

We present, to our knowledge, the most extensive application of machine learning to COVID-19 hospital survival in Brazil. We considered the very rich SIVEP-Gripe dataset as of September 21, 2020. We confirm several worldwide findings but also report important sociodemographic trends specific to Brazil.

We found that XGBoost outperforms other methods including logistic regression (Supplementary Materials, Section S3D). This improved performance demonstrates the non-linearity and co-linearity present in the data and justifies the choice of a machine learning model over conventional statistical techniques. The trained model is publicly available at https://github.com/PedroBaqui/XCOVID-BR.

Using XCOVID-BR we find that socioeconomic factors are more important than comorbidities (Figs. 4 and 5), a scenario that seems to reflect the social inequalities present throughout Brazil. The number of comorbidities remains, however, the third most important feature, signaling that the interaction between comorbidities is a significant factor for the outcome of COVID-19 patients. We also confirm that the patient’s age is the most important factor. It is worth noting, that age correlates with dementia, which has been shown to increase susceptibility to COVID-19^15^. We highlight the following factors: the state of residence and its development index, the distance to the hospital (very important for rural and less developed areas), the level of education, and hospital funding and strain. Social factors such as the level of education, correlated to income, are related to access to trustworthy information that may impact the susceptibility to COVID-19^16^. Our analysis, however, does not consider data from social media and news about COVID-19. Ethnicity is also confirmed to be more important than comorbidities in agreement with an earlier investigation that adopted mixed-effects Cox regression survival analysis^2^. Here, we also include socio-geographic features and model non-linear interactions via XGBoost and find that socio-geographic features are more important than ethnicity.

These findings qualitatively agree with the results from the descriptive and odds ratio analysis (Table 1): non-survivors are older, more likely to have been admitted to public hospitals and live in less developed cities. Survivors are more likely to be white Brazilians, with high/higher education, living in urban areas. We also confirm the higher proportion of non-survivors in the North and Northeast macro-regions^2^. Additionally, comorbidities, except for asthma, are more prevalent among non-survivors, especially renal and neurological diseases. The most common comorbidities were cardiovascular disease and diabetes.

Of course many of these variables are correlated: Patients with access to private healthcare tend to have a higher education and better living conditions (city development index). While the latter means that one shares a household with fewer people and the ready availability of basic services such as running water and sanitation, the former gives the possibility to work remotely. Poor literacy is likely to also impact negatively on healthcare access. These findings support the conclusion that socioeconomic, ethnic and geographical factors are crucial in order to correctly understand the pandemic in Brazil and plan adequate measures^17^.

We tested the predictive performance of the XCOVID-BR model for various sub-groups of the SIVEP-Gripe dataset (Table 1, AUC values) and found that the performance is generally similar to the global one, except for a few cases such as the North macro-region, illiterate Brazilians and some groups with comorbidities. The lower performance relative to these sub-groups indicates that it is more difficult to forecast the evolution of the disease within certain sections of Brazilian society, possibly because there is not enough data for these sub-groups which may be characterized by a higher heterogeneity. In other words, these groups are more susceptible to COVID-19, and it is also harder to study factors underlying their COVID-19 mortality risk. We hope this result will be useful in motivating federal authorities in adopting effective action in order to mitigate the impact of the pandemic for these groups.

Hospital funding model (private or public) was found to be a very important feature (Fig. 4). We indeed clearly observe that public healthcare suffers from a higher mortality rate across all ages (Fig. 6). This is not unexpected as private healthcare serves only 25% of the Brazilian population and total spending is similar to that of public healthcare, implying that, on average, a patient in a private hospital costs three times more than one in a public hospital^18^. In particular, public hospitals have 1.4 ICU beds per 10 thousand inhabitants, while private hospitals have 4.9. This difference is more pronounced in the North and Northeast regions, with 0.9 and 1.5 beds per 10 thousand inhabitants in public hospitals against 4.7 and 5.5 beds per 10 thousand inhabitants for private hospitals, respectively^19^. Complementary to a hospital’s funding is its level of strain. Our findings are in line with the findings of previous studies^20,21^ and suggest the importance of funding public hospitals and better managing the healthcare network, with profound implications for policy making in Brazil.

Finally, we showed how one can use the XCOVID-BR model in order to estimate the mortality risk of specific groups of the Brazilian population. In other words, one can apply XCOVID-BR to arbitrary sections of the Brazilian population and estimate the differential risk from COVID-19 (Fig. 1), helping policy makers to take informed decisions regarding vaccination/non-pharmacological preventative measures, hospital management and healthcare network organization in an equitable way.

As an example, we showed how the risk distribution differs between two representative areas: The wealthier Paraná and more socioeconomically challenged Pernambuco (Fig. 7). The variation in probability distributions is striking. Accounting for age, the dominant clinical predictor of mortality, it is apparent that the probability distribution is heavily skewed to higher probabilities of recovery in the wealthier (Paraná) region and this is particularly apparent in younger patients and in privately-funded hospitals.

Although we believe our work is the most comprehensive of its kind to date in Brazil, there are limitations which need discussion. Possible biases from case ascertainment cannot be ruled out, in common with all observational / retrospective database research. Data completeness was generally good, however. Because of our selection criteria (Fig. 2), data missingness is largely confined to ethnicity (9.0%), city type (10.5%) and education level (28.3%, see Supplementary Materials, Section S3C), values that are overall better or comparable to a recent large dataset from the UK (26% of data with missing ethnicity)^22^. We considered only patients who were hospitalized, since testing in the community is more likely to be biased according to local factors. However, a residual inhomogeneity in this population could skew our results according to local factors, even though this should be mitigated by the large number of diverse covariates we consider, and the use of the XGB model that can cope with nonlinear correlations.

Health-seeking behavior may vary across Brazil. First, late presentation may be an important determinant of hospital outcome. We could not address this directly as data for physiological severity at hospital presentation are not available, but we considered correlated socioeconomic and structural factors such as the distance to the hospital. Secondly, it is important to point out that we do not have data on out-of-hospital mortality, which may be substantial. As such, a consideration of hospital mortality is likely to underestimate the relative differences in risk factors, and it is plausible to assume that healthcare availability inequities would be further amplified in patients who are not hospitalized. In other words, it is reasonable to assume that socioeconomic and structural factors are even more important than the findings of this study might suggest. Urgent work is needed to better understand deaths occurring in the community.

The XGB model also suffers from a number of limitations, common to other machine learning models. First, our results, in particular feature importance, depend, to some extent, on the details of the numerical implementation. To assess this important aspect, we tested other feature importance methods (Supplementary Materials, Section S3E) and confirmed the higher importance of socio-geographical and hospital-specific features, as compared to comorbidities. Second, supervised machine learning models such as XGB connect features to outcome and their success is tied to the dataset on which they are trained. Consequently, the previously discussed dataset limitations are also the limitations of our XGB model.

The current vaccination plan proposed by the Brazilian Ministry of Health^23^ closely follows the plans devised by countries in Europe such as the UK^24^. In particular, prioritization is mostly based on age and comorbidities. While these factors are undoubtedly significant, we have shown here that in Brazil they are not the sole risk factors and that socioeconomic and structural factors are actually as important in order to reduce COVID-19 mortality^25–27^. Based on our findings, we recommend that the Brazilian Ministry of Health should adopt vaccination/non-pharmacological preventative measures that are properly tailored to the complex socioeconomic profile of Brazil. Specifically, we recommend boosting the resources of strained public hospitals, facilitating access to medical care, and targeting the socio-geographic sections of Brazil that are less economically developed.

Finally, given the changing nature of the virus, with ever more frequent emergence of SARS-CoV-2 variants, it is worth stressing the significance of data-driven risk factor discovery. Indeed, one expects that the relative importance of biological and structural COVID-19 risk factors depends on case fatality rate, transmissibility and response to vaccination efforts of the new variants. A data driven approach seems to be an agile approach to understand such an ever-changing scenario.

## Supplementary Information

Supplementary Information.

## Data Availability

The data that support the findings of this study are available from the corresponding author upon reasonable request. SIVEP-Gripe data are publicly available at https://opendatasus.saude.gov.br/dataset/bd-srag-2020. Our analysis code and XCOVID-BR are available at https://github.com/PedroBaqui/XCOVID-BR.

## References

[1] Worldometers. Brazil. Web page https://www.worldometers.info/coronavirus/country/brazil/. Accessed 19 June 2021 (2021).

[2] Baqui P, Bica I, Marra V, Ercole A, van der Schaar M (2020). Ethnic and regional variations in hospital mortality from COVID-19 in Brazil: A cross-sectional observational study. Lancet Global Health.

[3] Barberia LG, Gómez EJ (2020). Political and institutional perils of Brazil’s COVID-19 crisis. Lancet.

[4] Docherty AB (2020). Features of 20 133 UK patients in hospital with Covid-19 using the isaric who clinical characterisation protocol: prospective observational cohort study. BMJ.

[5] Nachtigall I (2020). Clinical course and factors associated with outcomes among 1904 patients hospitalized with covid-19 in germany: An observational study. Clin. Microbiol. Infect..

[6] Grasselli G (2020). Baseline Characteristics and Outcomes of 1591 Patients Infected With SARS-CoV-2 Admitted to ICUs of the Lombardy Region. Italy. JAMA.

[7] Marra, V. & Quartin, M. A Bayesian estimate of the COVID-19 infection fatality ratio in Brazil based on a random seroprevalence survey. medRxiv 10.1101/2020.08.18.20177626 (2021). 10.1016/j.ijid.2021.08.016 PMC8358085 34390858

[8] Qian Z, Alaa AM, van der Schaar M, Ercole A (2020). Between-centre differences for covid-19 icu mortality from early data in england. Intensive Care Med..

[9] Bravata DM (2021). Association of Intensive Care Unit Patient Load and Demand With Mortality Rates in US Department of Veterans Affairs Hospitals During the COVID-19 Pandemic. JAMA Network Open.

[10] Taccone FS (2021). The role of organizational characteristics on the outcome of covid-19 patients admitted to the icu in belgium. Lancet Reg. Health Europe.

[11] Ministry of Health. SRAG 2020. Web page https://opendatasus.saude.gov.br/dataset/bd-srag-2020. Accessed 5 Oct 2020 (2020).

[12] Pedregosa F (2011). Scikit-learn: Machine Learning in Python. J. Mach. Learn. Res..

[13] Chen, T. & Guestrin, C. Xgboost: A scalable tree boosting system. In Proceedings of the 22nd ACM SIGKDD International Conference on Knowledge Discovery and Data Mining, KDD’16, 785–794, 10.1145/2939672.2939785 (Association for Computing Machinery, New York, NY, USA, 2016).

[14] Collins GS, Reitsma JB, Altman DG, Moons KG (2015). Transparent Reporting of a multivariable prediction model for Individual Prognosis Or Diagnosis (TRIPOD): The TRIPOD Statement. Ann. Internal Med..

[15] Hariyanto TI, Putri C, Arisa J, Situmeang RFV, Kurniawan A (2021). Dementia and outcomes from coronavirus disease 2019 (covid-19) pneumonia: A systematic review and meta-analysis. Arch. Gerontol. Geriatrics.

[16] Halim DA (2020). Understanding of young people about covid-19 during early outbreak in indonesia. Asia Pac. J. Public Health.

[17] Ribeiro H, Lima VM, Waldman EA (2020). In the COVID-19 pandemic in Brazil, do brown lives matter?. Lancet Global Health.

[18] de Carvalho, T. Saúde Pública: um panorama do Brasil. Web page https://www.politize.com.br/panorama-da-saude/. Accessed 11 Jan 2021 (2018).

[19] Associação de Medicina Intensiva Brasileira. AMIB apresenta dados atualizados sobre leitos de UTI no Brasil. Web page https://www.amib.org.br/fileadmin/user_upload/amib/2020/abril/28/dados_uti_amib.pdf. Accessed 29 July 2020 (2020).

[20] Wilde, H. *et al.* The association between mechanical ventilator availability and mortality risk in intensive care patients with COVID-19: A national retrospective cohort study. medRxiv 10.1101/2021.01.11.21249461 (2021). 10.1186/s12916-021-02096-0 PMC8404408 34461893

[21] Rubinson L (2021). Intensive Care Unit Strain and Mortality Risk Among Critically Ill Patients With COVID-19-There Is No Me in COVID. JAMA Netw. Open.

[22] Williamson EJ (2020). Factors associated with covid-19-related death using opensafely. Nature.

[23] Ministry of Health. Plano nacional de operacionalização da vacinação contra a COVID-19. Web page https://www.gov.br/saude/pt-br/media/pdf/2020/dezembro/16/plano_vacinacao_versao_eletronica-1.pdf. Accessed 11 Jan 2021 (2020).

[24] Joint Committee on Vaccination and Immunisation. Priority groups for coronavirus (COVID-19) vaccination. Web page https://www.gov.uk/government/publications/priority-groups-for-coronavirus-covid-19-vaccination-advice-from-the-jcvi-30-december-2020. Accessed 11 Jan 2021 (2020).

[25] Hassan-Smith Z, Hanif W, Khunti K (2020). Who should be prioritised for COVID-19 vaccines?. Lancet.

[26] Richards-Belle A (2020). COVID-19 in critical care: epidemiology of the first epidemic wave across England, Wales and Northern Ireland. Intensive Care Med..

[27] Campos-Matos I (2021). Maximising benefit, reducing inequalities and ensuring deliverability: Prioritisation of COVID-19 vaccination in the UK. Lancet Reg. Health Europe.

