# Supplementary material for: Comparing COVID-19 risk factors in Brazil using machine learning: the importance of socioeconomic, demographic and structural factors
Source: Sci Rep. 2021 Aug 2;11:15591. doi: 10.1038/s41598-021-95004-8 (PMC8329284; doi:10.1038/s41598-021-95004-8)
Supplement: Supplementary file 1 — Supplementary Information. [file 41598_2021_95004_MOESM1_ESM.pdf]

Supplementary materials
for
Comparing COVID-19 risk factors in Brazil using machine learning: the importance of socioeconomic, demographic and structural factors

Pedro Baqui,[1] Valerio Marra,[1,2] Ahmed M. Alaa,[3] Ioana Bica,[4,5] Ari Ercole,[6,7] and Mihaela van der Schaar[3,5,7,8]

[1] *Núcleo de Astrofísica e Cosmologia, Universidade Federal do Espírito Santo, Vitória, ES, Brazil*
[2] *Departamento de Física, Universidade Federal do Espírito Santo, Vitória, ES, Brazil*
[3] *Department of Electrical and Computer Engineering, University of California Los Angeles, Los Angeles, CA, USA*
[4] *Department of Engineering Science, University of Oxford, Oxford, UK*
[5] *The Alan Turing Institute, London, UK*
[6] *Department of Medicine, University of Cambridge, Cambridge, UK*
[7] *Cambridge Centre for Artificial Intelligence in Medicine, Cambridge, UK*
[8] *Department of Applied Mathematics and Theoretical Physics and Department of Population Health, University of Cambridge, Cambridge, UK*

CONTENTS

S1. Geographical spread of SARS-CoV-2 1

S2. Differences among the macro-regions 4

S3. Machine learning details 4
  A. Adopted features 4
  B. Hyperparameters 5
  C. Handling of missing values 5
  D. Machine learning performance 5
  E. Feature importance robustness 5

References 5

S1. GEOGRAPHICAL SPREAD OF SARS-COV-2

Brazil is divided into 27 Federative Units (26 states and 1 federal district), which are grouped into five macro-regions:

- North: Rondônia (RO), Acre (AC), Amazonas (AM), Roraima (RR), Pará (PA), Amapá (AP), Tocantins (TO);
- Northeast: Bahia (BA), Piaui (PI), Maranhão (MA), Ceará (CE), Rio Grande do Norte (RN), Paraíba (PB), Pernambuco (PE), Alagoas (AL), Sergipe (SE);
- Central-West: Distrito Federal (DF), Goiás (GO), Mato Grosso (MT), Mato Grosso do Sul (MS);
- Southeast: São Paulo (SP), Rio de janeiro (RJ), Espírito Santo(ES), Minas Gerais (MG);
- South: Santa Catarina (SC), Paraná (PR), Rio Grande do Sul (RS).

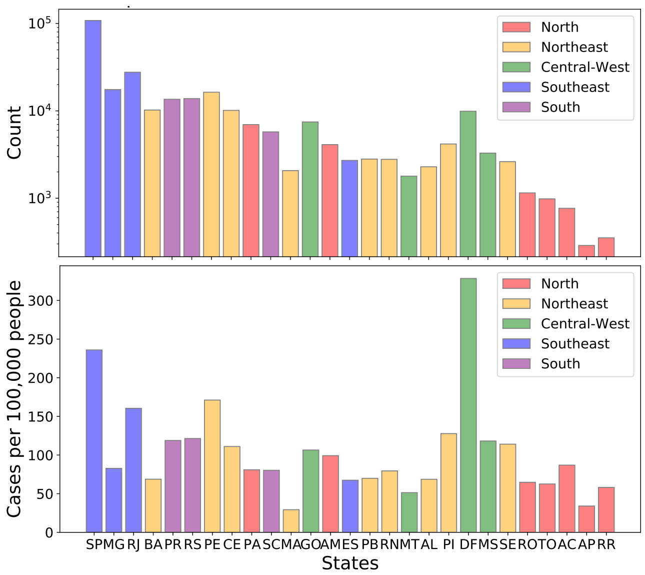

Figure S1. **Distribution of the 279,987 hospitalized patients with SARS-CoV-2 among Brazilian states according to absolute number of cases and number of cases per 100,000 people.** The different colors represent the Brazilian macro-regions. States are ordered according to their population, larger on the left.

Figure S1 shows the distribution of hospitalized SARS-CoV-2 patients, colored according to macro-region. By comparing the distribution of hospitalized SARS-CoV-2 patients among the Brazilian states with earlier investigations (Figure S1),[1] it is evident that the pandemic propagated though Brazil, affecting basically all the states. This indicates a failure in non-pharmaceutical interventions such as social distancing.

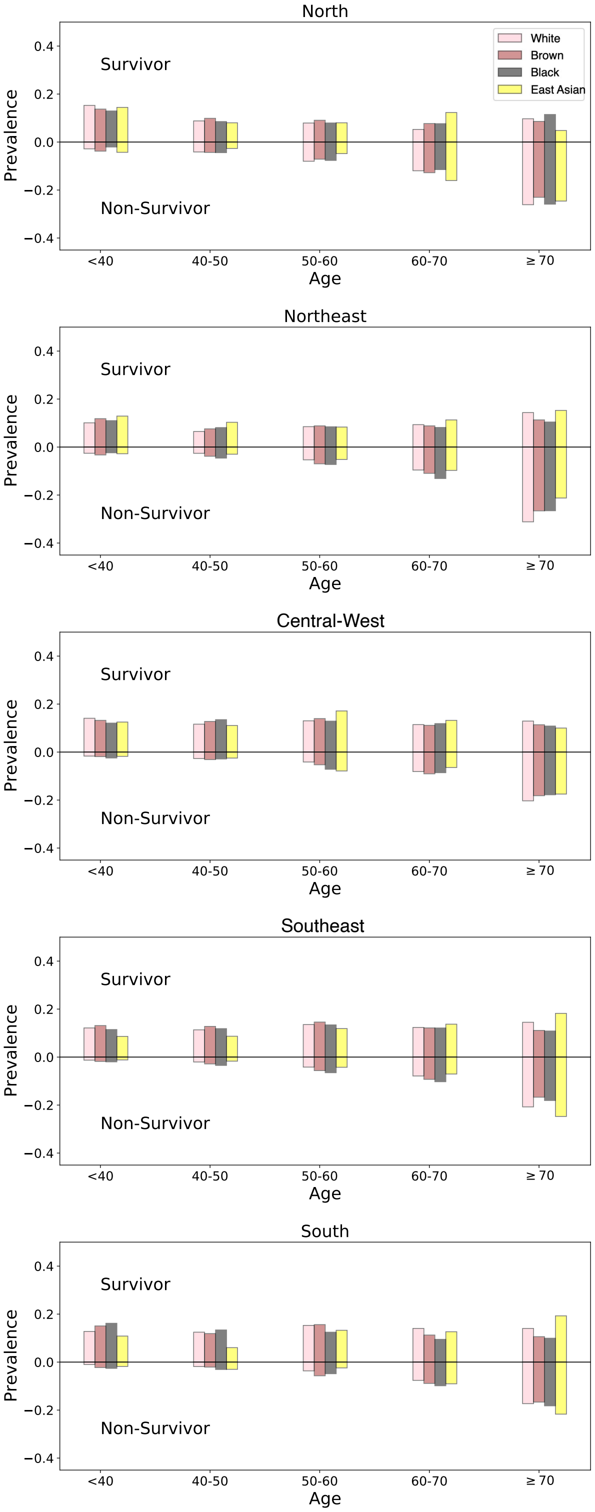

Figure S2. **Distributions of ethnicity according to age.** The normalization is such that all the fractions of a given ethnicity add to unity (to adjust for differences in ethnic prevalence). We exclude Indigenous patients for clarity because of their small numbers in the study population.

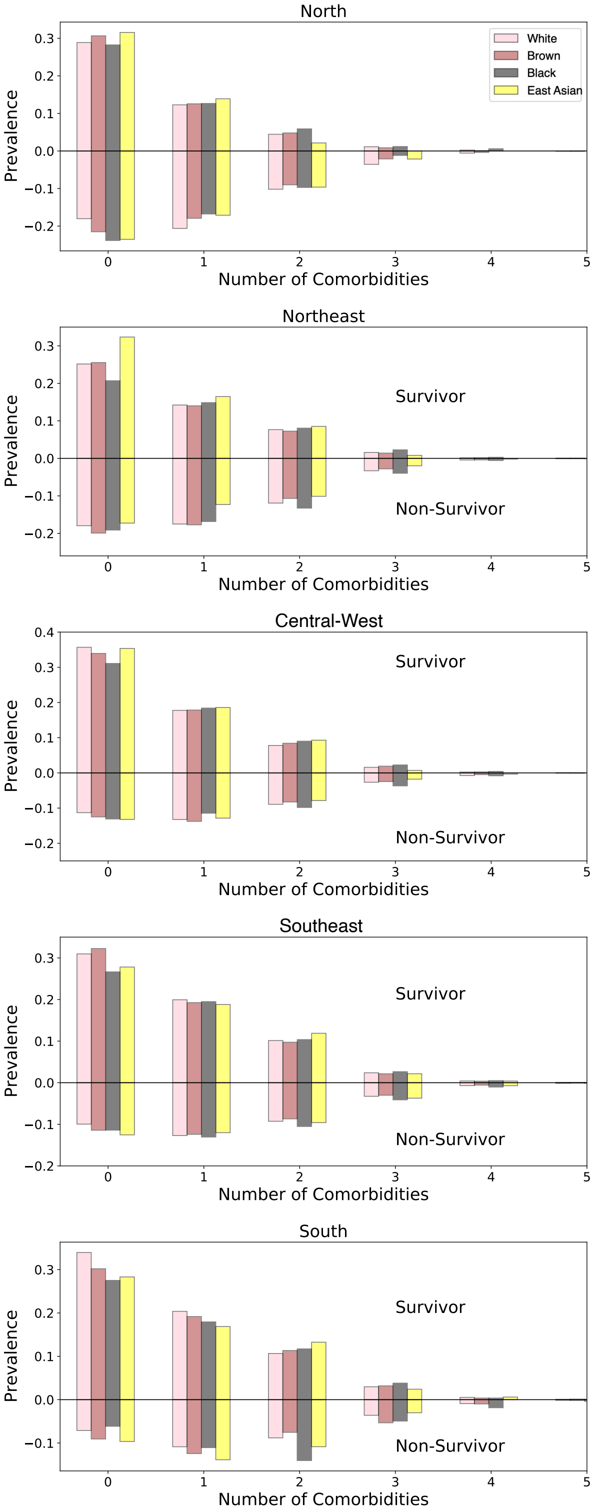

Figure S3. **Distributions of ethnicity according to number of comorbidities.** The normalization is such that all the fractions of a given ethnicity add to unity. We exclude Indigenous patients for clarity because of their small numbers in the study population.

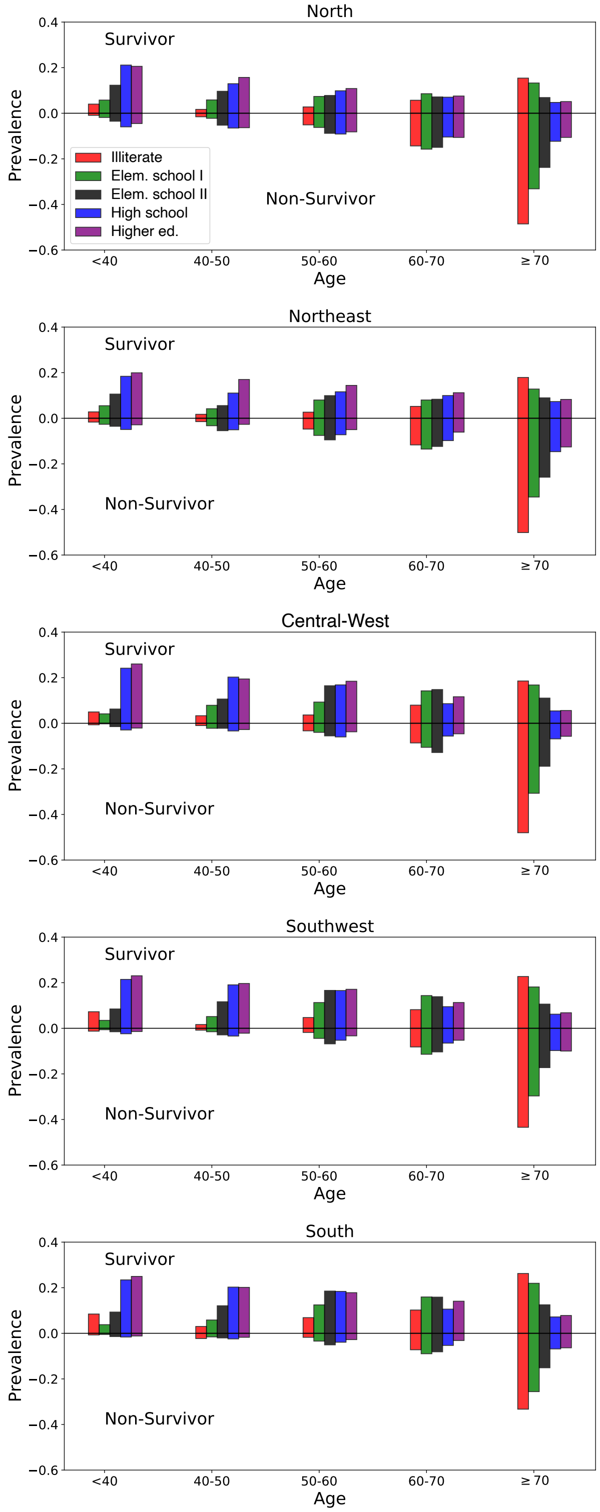

Figure S4. **Distributions of education level according to age.** The normalization is such that all the fractions of a given education level add to unity.

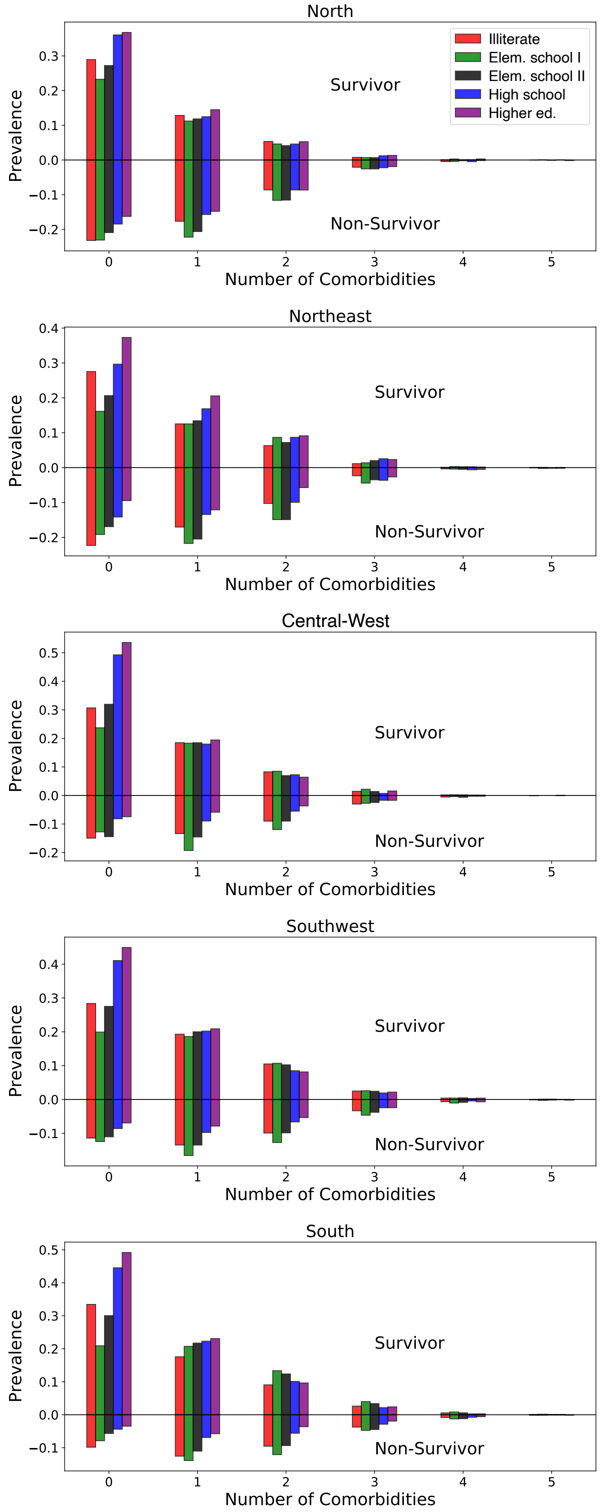

Figure S5. **Distributions of education level according to number of comorbidities.** The normalization is such that all the fractions of a given education level add to unity.

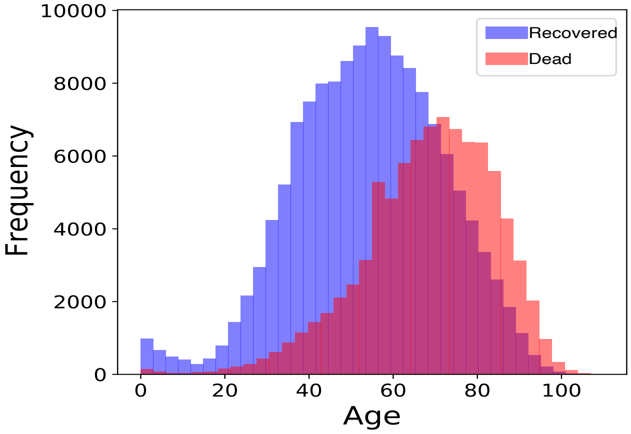

Figure S6. **Age distribution of the hospitalized patients considered in this work.**

S2. DIFFERENCES AMONG THE MACRO-REGIONS

Brazil is a large and diverse country. Although each state has its individual particularities, each macro-region shares among its members socioeconomic similarities. Figures S2-S5 give an overall view of the differences among the five Brazilian macro-regions. The common trend is that the South and Southeast regions have responded better to the pandemic compared to the North and Northeast regions. The figures are normalized so that the sum of each ethnicity is equal to one, independently. In this analysis we used data from 242,679 patients, relaxing some constraints on municipality and hospital data.

Figures S2-S3 are stratified according to ethnicity as the latter is correlated with poverty.[2] Poverty implies a higher lever of susceptibility to SARS-CoV-2 as remote working may not be possible and poor people tend to live in crowed households with less access to sanitation.[3] Moreover, they do not have access to private health care. Figures S4-S5 are stratified according to education, which is again correlated with poverty. Illiteracy refers to patients without education and more than seven years of age. As shown by Figure S6, our dataset includes mostly adult patients.

S3. MACHINE LEARNING DETAILS

A. Adopted features

For completeness we list below the features that we considered when implementing the machine learning algorithms:

- **Clinical Factors**: age, sex, ethnicity, comorbidities (cardiovascular disease, liver disease, asthma, pulmonary disease, renal disease, hematologic disease, diabetes, obesity, neurological disease, immunosuppression, sum of comorbidities), symp-

Table S1. Percentages of patients with missing values.

Feature	No. (%)
Age	0 (0.0%)
Sex	0 (0.0%)
Funding Model	0 (0.0%)
MHDI	0 (0.0%)
Ethnic group	20877 (9.0%)
Macro-region	0 (0.0%)
City type	24353 (10.5%)
Education level	65366 (28.3%)
Comorbidities	
Cardiovascular disease	104841 (45.4%)
Asthma	132906 (57.5%)
Diabetes	112748 (48.8%)
Pulmonary disease	131744 (57.0%)
Obesity	131784 (57.0%)
Immunosuppression	132993 (57.5%)
Renal disease	131622 (57.0%)
Liver disease	134321 (58.1%)
Neurological disease	131494 (56.9%)
Hematologic disease	133999 (58.0%)
Symptoms	
Fever	21277 (9.2%)
Vomiting	57379 (24.8%)
Cough	18765 (8.1%)
Sore throat	51474 (22.3%)
Respiratory discomfort	34119 (14.8%)
Shortness breath	20421 (8.8%)
Diarrhea	54109 (23.4%)
SpO2 < 95%	30981 (13.4%)

Model	AUC (95%CI)	$AP_{recovery}$ (95%CI)	AP_{death} (95%CI)
**XGB**	**0.813** [0.810, 0.817]	**0.879** [0.876, 0.883]	**0.711** [0.704, 0.721]
**XGB***	**0.797** [0.793, 0.801]	**0.866** [0.863, 0.870]	**0.689** [0.682, 0.698]
RF	0.798 [0.793, 0.803]	0.867 [0.863, 0.871]	0.686 [0.678, 0.696]
RF*	0.781 [0.778, 0.786]	0.854 [0.851, 0.859]	0.661 [0.654, 0.669]
NN	0.795 [0.791, 0.800]	0.865 [0.861, 0.869]	0.677 [0.670, 0.685]
NN*	0.782 [0.779, 0.786]	0.855 [0.852, 0.859]	0.660 [0.653, 0.670]
LR	0.766 [0.761, 0.770]	0.840 [0.835, 0.845]	0.632 [0.622, 0.640]
LR*	0.763 [0.759, 0.768]	0.837 [0.833, 0.842]	0.629 [0.619, 0.639]
SVM	0.766 [0.761, 0.770]	0.842 [0.838, 0.847]	0.635 [0.627, 0.644]
SVM*	0.761 [0.757, 0.766]	0.838 [0.834, 0.843]	0.628 [0.619, 0.638]
KNN	0.764 [0.760, 0.769]	0.834 [0.830, 0.839]	0.634 [0.627, 0.642]
KNN*	0.751 [0.746, 0.756]	0.825 [0.820, 0.829]	0.615 [0.607, 0.622]

Table S2. **Performance of the machine learning algorithms considered in this work.** The analyses with "*" sign do not consider symptoms.

toms (fever, vomiting, cough, sore throat, respiratory discomfort, shortness of breath, diarrhea, SpO2<95%, sum of symptoms).

- **Socio-Geographic**: education, state, MHDI, city type, distance to hospital.

- **Health System**: funding (private or public), strain.

In total we considered 30 features.

B. Hyperparameters

The hyper-parameters adopted for XGBoost are:

```
n_estimators: 200
eta: 0.2
max_depth: 4
gamma: 1
subsample: 0.9
colsample_bytree: 0.9
```

More at github.com/PedroBaqui/XCOVID-BR.

C. Handling of missing values

The SIVEP-Gripe catalog has missing values. In the case of comorbidities or symptoms we imputed missing values as the clinical feature being absent for the individual.[1] For the remaining variables we did not perform pre-processing for the XGB algorithm as the latter already imputes missing data. On the other hand, for the LR, KNN, NN, RF and SVM models we adopted scikit-learn's SimpleImputer. Table S1 shows the percentages of patients with missing values for all the features that we consider.

D. Machine learning performance

Table S2 shows the performance of the machine learning algorithms considered in this work. The XGBoost algorithm achieves an excellent score and is adopted in the analysis of the main text.

E. Feature importance robustness

The result of the feature importance analysis may depend on the specific method adopted. Here, in order to test the robustness of our results, we consider different feature importance methods for the XGB algorithm without symptom information.

The result is shown in Figure S7 which should be compared to Figure 4 of the main text. The *Total Gain* method adopts gain values associated with the features that divided the data. The *Weight* method uses the number of times a particular feature divided the data. Finally, the *Total Cover* method makes use of the number of data points affected by the cut where a feature was used. The *Permutation* method adopted in Figure 4 of the main text randomly breaks the relationship between feature and target and measures the decrease in the metric used.[4]

We note that the various methods all give a higher importance to socio-geographical and hospital-specific features compared to comorbidities. In particular, hospital strain is confirmed to be a very important factor.

---

[1] Baqui P, Bica I, Marra V, Ercole A, and van der Schaar M. Ethnic and regional variations in hospital mortality from COVID-19 in Brazil: a cross-sectional observational study. *The Lancet Global Health*, 2020; **8**: 1018–26.

[2] IBGE. Desigualdades Sociais por Cor ou Raça no Brasil, 2019. biblioteca.ibge.gov.br/visualizacao/livros/liv101681_informativo.pdf. (accessed July 27, 2020).

[3] Tavares F and Betti G. Vulnerability, Poverty and COVID-19: Risk Factors and Deprivations in Brazil, 2020. researchgate.net/publication/340660228_Vulnerability_Poverty_and_COVID-19_Risk_Factors_and_Deprivations_in_Brazil (accessed May 10, 2020).

[4] xgboost developers. Python API Reference, 2020. xgboost.readthedocs.io/en/latest/python/python_api.html. (accessed August 23, 2020).

[5] Collins GS, Reitsma JB, Altman DG, and Moons KG. Transparent Reporting of a multivariable prediction model for Individual Prognosis Or Diagnosis (TRIPOD): The TRIPOD Statement. *Annals of Internal Medicine*, 2015; **162**: 55–63.

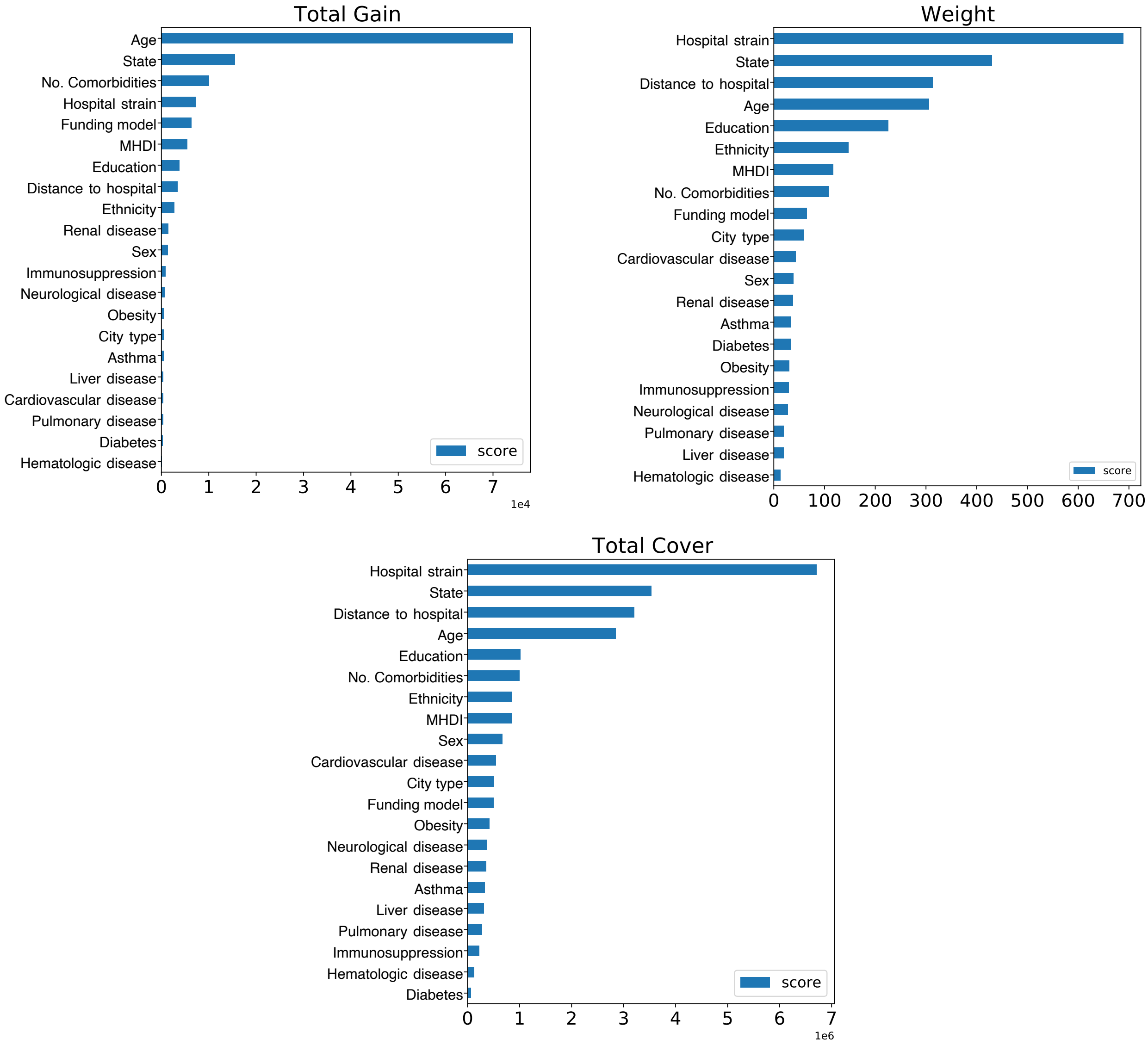

Figure S7. Different methods used to calculate feature importance for the XGB algorithm over the training set.
